# PARALLEL-VT: A pilot study of simultaneous multi-wavefront left ventricular mapping for identification of critical ablation sites

**DOI:** 10.1007/s10840-026-02347-1

**Published:** 2026-05-06

**Authors:** Jakub Sroubek, Mandeep Bhargava, Koji Higuchi, Ayman A. Hussein, Niraj Varma, Oussama M. Wazni, Elad Anter

**Affiliations:** 1 Section of Cardiac Pacing and Electrophysiology, Department of Cardiovascular Medicine, Cleveland Clinic, 9500 Euclid Avenue, Cleveland, OH 44195 USA; 2 Department of Cardiology, Shamir Medical Center, Tel Aviv, Israel

**Keywords:** Ventricular tachycardia, Catheter ablation, Radiofrequency Ablation, Substrate Mapping, Cardiomyopathy

## Abstract

**Background:**

Functional substrate mapping can identify ventricular tachycardia (VT) ablation targets. Multi-wavefront functional mapping can be laborious. In this study, we explored the feasibility, safety and utility of the new “parallel mapping” algorithm designed to streamline multi-wavefront activation mapping for VT.

**Methods:**

This is a single center, prospective pilot study of adults undergoing scar-related VT ablation. Patients underwent endocardial and/or epicardial substrate parallel mapping using 2 distinct activation wavefronts (e.g. right ventricular [RV] and left ventricular [LV] pacing, 8 beats each). Slow-conducting regions were targeted for radiofrequency ablation. Short- and long-term outcomes were analyzed.

**Results:**

Of the 41 ablation subjects, 19 (46.3%) underwent per-protocol parallel mapping. Protocol deviations mostly stemmed from incessant VT necessitating early ablation (*n* = 9, 40.9%). Most per-protocol treated subjects were male (*n* = 18, 94.7%) with a mean age of 66 ± 10 years and had a higher prevalence of ischemic heart disease compared to protocol deviations (94.7% vs. 54.6%, respectively, *p* = 0.005). Primary wavefront mapping was done during RV apical pacing. Secondary wavefront mapping chiefly relied on LV pacing (52.6%). The median procedure duration was 252 min (IQR 190–289). Adverse events occurred in 4 patients (21.1%; at most 1 event directly related to study protocol). In the first year of follow-up, seven (36.8%) individuals received appropriate ICD shocks and three (15.8%) patients died. In exploratory analyses, parallel mapping data appeared most informative in subjects with lateral wall scar.

**Conclusion:**

Parallel substrate mapping using multiple activation wavefronts is a novel, feasible and safe algorithm designed to identify ablation targets in patients with scar-related VT.

**Graphical Abstract:**

**Figure:**
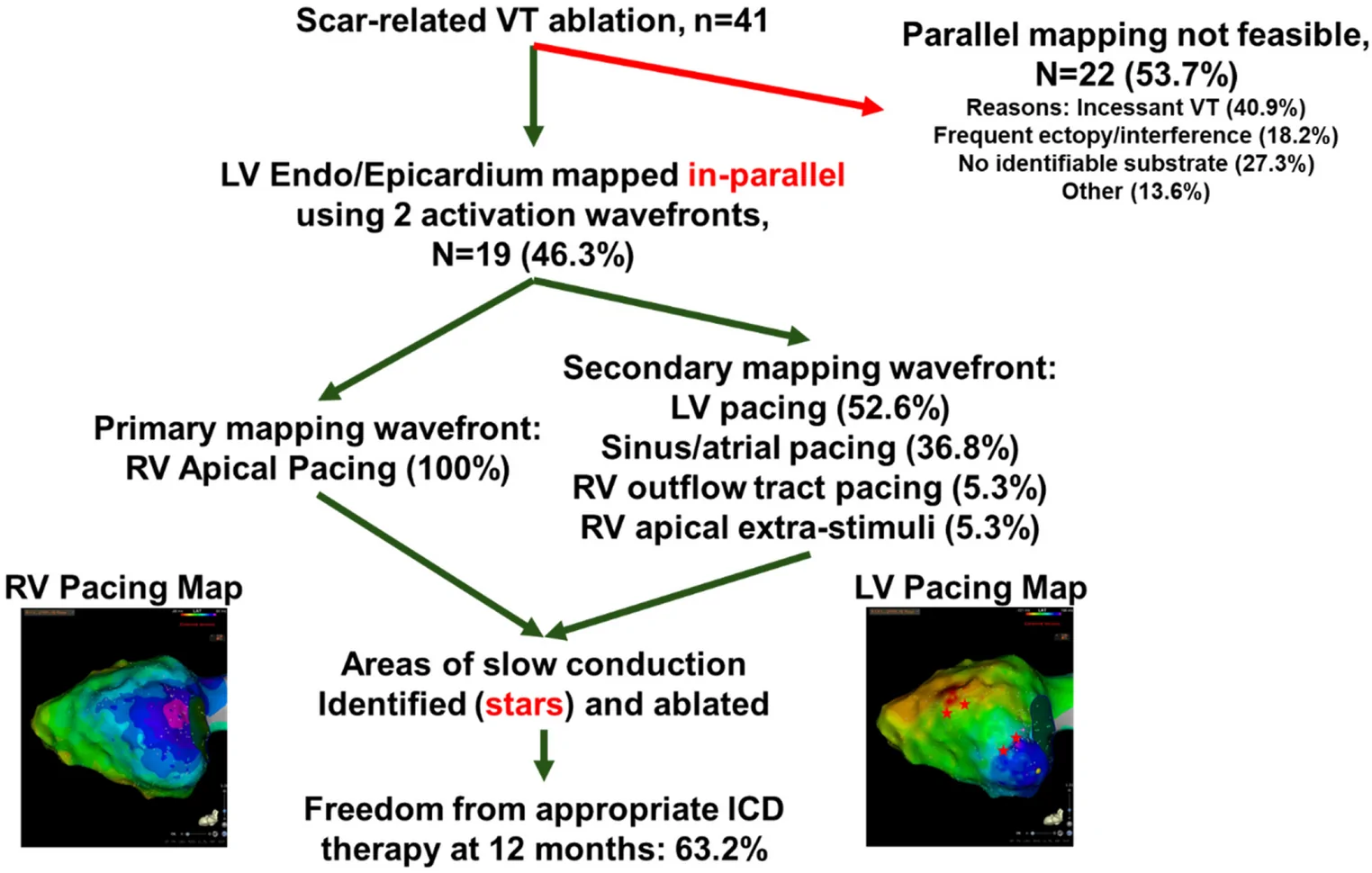
Outline of the parallel substrate mapping pilot study. ICD, implantable cardioverter-defibrillator; LV = left ventricle; RV = right ventricle

**Supplementary Information:**

The online version contains supplementary material available at https://doi.org/10.1007/s10840-026-02347-1.

## Introduction

Management of scar-related ventricular tachycardia (VT) often relies on catheter ablation, a potentially curative treatment. Despite technological advances, the efficacy of ablation remains limited in certain cases, particularly when comprehensive activation mapping of all suspected VT circuits is not feasible. As a result, the past two decades have seen the development of substrate-based mapping techniques, which allow identification of ablation targets during stable, non-VT rhythms. Traditionally, these techniques focus on detecting abnormal electrograms, including low-voltage signals, fractionated or late potentials [1, 2], and regions of slow conduction [3]. 

A common limitation of substrate mapping techniques is their reliance on mapping during a single type of rhythm (e.g. sinus rhythm only), which may not accurately reflect the substrate during VT. During VT, differences in wavefront direction and cycle length can alter conduction patterns, potentially unmasking areas of abnormal conduction and repolarization that may not be evident during sinus rhythm. As a result, critical sites involved in VT circuits may go undetected with conventional substrate mapping techniques.

To address this potential limitation, newer mapping techniques have been developed that assess the substrate under varying activation wavefronts and different cycle lengths. Changes in wavefront direction are achieved through sequential mapping during pacing from different sites, such as the right ventricle (RV) and the left ventricle (LV). Variations in cycle length are introduced using extrastimuli to evaluate conduction properties at shorter intervals—a method known as decremental evoked potential (DEEP) mapping [4]. Alternatively, areas of conduction slowing are identified during straight pacing from multiple wavefronts [5, 6]. Collectively, these are referred to as functional mapping techniques as they stress the myocardial conduction system to uncover the dynamic characteristics of the substrate. A key limitation of these techniques is their relatively complex workflow, the prolonged mapping time required, and the potential for hemodynamic instability during ventricular pacing. These factors may have contributed to their limited adoption in routine clinical practice.

Recently, a new software tool called “parallel mapping” was developed and integrated into the CARTO3 mapping system. This tool enables automatic and simultaneous creation of multiple activation maps. For example, during pacing at a stable cycle length of 600ms with a single extrastimulus at 300ms, the system can generate two maps concurrently, one for the S1 beat and one for the S2 beat. Alternatively, a pacing protocol that oscillates between two pacing sites, such as four beats from the RV followed by four beats from the LV (via a second catheter positioned in the LV cavity or in a ventricular branch of the coronary sinus) allows for simultaneous mapping of the LV from two different wavefront directions.

The aim of this pilot study was to assess the feasibility, utility and safety of the parallel mapping software, integrated into the CARTO3 system, for generating simultaneous substrate maps during VT ablation. Specifically, the study evaluated whether the software could produce maps from multiple activation wavefront directions or different cycle lengths in parallel, to streamline the substrate mapping process and enhance procedural efficiency.

## Methods

### Study design

This was a single-center, single-arm pilot feasibility study. All data were collected prospectively. The study was approved by the Institutional Review Board of the Cleveland Clinic. All patients signed an informed consent for the study.

### Patients

Patients were eligible for inclusion if they were ≥ 18 years of age, had a history of scar-mediated sustained ventricular tachycardia (VT), and were scheduled to undergo VT ablation as part of their standard clinical care. Exclusion criteria included an estimated life expectancy of less than one-year, current participation in another investigational drug or device study, or inability to understand the study and its requirements.

### Pre-operative management

When feasible, oral anticoagulation was withheld for ≥ 48 h prior to the procedure, and unfractionated heparin was discontinued for ≥ 6 h before the procedure. Anti-arrhythmic medications were held for ≥ 3 days prior to VT ablation with amiodarone discontinued for > 2 weeks whenever possible, unless the patient was in VT storm.

Based on the clinical judgement of the treating physician, patients deemed to be at very high risk for periprocedural hemodynamic decompensation were referred for placement of an Impella 5.0 or 5.5 device 24–48 h prior to the ablation procedure. The device was inserted via a surgical cut-down of the right axillary artery by the cardiac surgery team. Impella removal was planned within 24 h following VT ablation.

### Mapping protocol

The LV was mapped using Pentaray or DecaNav multipolar catheters and the CARTO3 mapping system (software version 7.5; Biosense Webster, Irvine, CA). Two distinct activation wavefronts were simultaneously acquired using the parallel mapping tool. The possible wavefronts included sinus rhythm (or atrial pacing), RV apical pacing, RV outflow tract pacing, and LV pacing via the coronary sinus (CS) lead in patients with a cardiac re-synchronization therapy (CRT) device, a temporary catheter placed in a ventricular branch of the CS or a catheter placed directly in contact with LV endocardium. The choice of pacing sites was based on the location of the scar, with the goal of stimulating close to the scar and from two orthogonal directions relative to the scar. LV endocardial catheter stability was tracked by the mapping system (based on catheter position and paced QRS morphology). The selection of pacing wavefronts ultimately reflected individual operator’s preference and procedural feasibility.

Pacing was performed using the Micropace stimulator (Micropace EP Inc, Santa Ana, CA) with a protocol that alternated stimulation between channels every 5 beats or more (Supplemental Material). All but the 1st beat in the drive train were used for mapping. In some cases, an additional parallel map was generated using RV extrastimulus pacing. For this, the RV was paced at a constant cycle length of 400–800ms (S1), with an extrastimulus (S2) delivered every 4 to 8 beats at a coupling interval 30–40ms above the ventricular effective refractory period (VERP). When extra-stimuli were employed, a 2,000–3,000ms pause was employed between pacing cycles. The final selection of pacing protocols was at the discretion of the primary operator.

Activation data were displayed as isochronal maps in 10ms increments. Areas of activation slowing, defined as activation speed ≤ 25 cm/s (i.e., ≥ 4 isochrones within a 10 mm region), were identified and labeled as reentry-vulnerable zones (RVZs). Local activation timing was annotated automatically using the wavefront algorithm [7]. When mapping produced internally inconsistent results, individual datapoints were manually adjudicated for inclusion/exclusion.

VT induction was performed using programmed electrical stimulation. Hemodynamically stable VTs were mapped, and critical sites were identified through activation and/or entrainment mapping.

### Ablation protocol

Ablation targets included all RVZs identified during substrate mapping acquired under different activation wavefronts or cycle lengths using the parallel mapping tool, as well as all critical VT sites identified through activation or entrainment mapping during VT.

Radiofrequency ablation (RFA) was delivered using an irrigated, contact force-sensing catheter (ThermoCool ST SF, Biosense Webster, Irvine, CA) at 30–50 W for up to 120 s per lesion. Repetition of programmed ventricular stimulation was encouraged at the end of the procedure. If sustained monomorphic VT remained inducible, additional ablation was performed until non-inducibility was achieved, provided the patient remained hemodynamically stable. Additional ablation targets included previously identified substrate (i.e. additional ablation in the same area as before) or were based on additional substrate mapping and/or VT activation mapping, per operator’s judgement.

### Mapping data-analysis

Mapping data were analyzed retrospectively using the CARTO3 workstation. For each patient, voltage and activation maps acquired under different activation wavefronts or cycle lengths were compared offline using consistent bipolar voltage thresholds and identical isochronal step settings.

### Follow-up

All patients who underwent per-protocol mapping and ablation were followed prospectively. Follow-up included scheduled visits every 3 months for the first year and additional visits as clinically indicated thereafter. Follow-up assessments included clinical history, physical examination, and ICD interrogation.

### Outcome measures

As a pilot study, the primary focus was to assess the feasibility and safety of the parallel mapping-guided VT ablation strategy. Feasibility was determined by the proportion of consented patients in whom full protocol-compliant parallel mapping could be performed. Reasons for post-consent exclusion were reviewed. Among per-protocol managed patients, procedural characteristics, safety outcomes, and efficacy were evaluated. These included total procedure time, fluoroscopy duration, complications, and the incidence of appropriate ICD shocks during follow-up. Procedural workflow, including impact of mapping/pacing strategies on the rate of data acquisition was also reviewed.

### Statistical analysis

Descriptive variables were summarized using means and standard deviations (SDs), medians and interquartile ranges (IQRs), or frequencies, as appropriate. Differences between groups were assessed using Fisher’s exact test, Student’s *t-test*, or the Wilcoxon rank-sum test. Freedom from appropriate ICD shocks was analyzed using Kaplan-Meier survival estimates. All statistical analyses were performed using StataMP 16 (StataCorp, College Station, TX).

## Results

### Patients

A total of 44 patients signed the informed consent form, but 2 never underwent a catheter ablation due to an unexpected change in their clinical management plan; 1 patient did undergo a VT ablation procedure which, for logistical reasons, had to be done using a non-CARTO3 mapping system. These 3 patients were considered immediate screen failures and were excluded from analysis.

Of the other 41 individuals who entered the EP lab for the procedure using the expected mapping system, 19 (46.3%) underwent per-protocol parallel mapping. The remaining 22 (53.7%) patients did not complete the protocol for the following reasons: ongoing VT (spontaneous or due to catheter ectopy) necessitating ablation before substrate mapping (*n* = 9, 40.9%), frequent ectopy and/or interference from an Impella device (*n* = 4, 18.2%), absence of identifiable substrate (*n* = 6, 27.3%: in all cases, initial limited substrate mapping revealed lack of abnormal potentials leading to a decision to switch to a different mapping/ablation strategy; in 1 case, focal papillary VT was suspected), and operator preference that was not otherwise specified in post-procedure review (*n* = 3, 13.6%).

Patients who completed the parallel mapping protocol were significantly more likely to have ischemic heart disease compared to those who did not (94.7% vs. 54.6%, respectively; *p* = 0.005). Conversely, there was a trend toward higher prevalence of advanced heart failure symptoms (New York Heart Association [NYHA] class III-IV) among patients who deviated from the investigational protocol than those who completed the study (36.4% vs. 10.5%, respectively; *p* = 0.075). No other significant differences were observed between the two groups (Table 1).

**Table 1:** Baseline patient characteristics

Variable	Using Parallel Mapping	Not Using Parallel Mapping	*P*-Value
Age, mean ± SD	66 ± 10	65 ± 10	0.312
Male, n (%)	18 (94.7)	20 (90.9)	1.000
Diabetes, n (%)	9 (47.4)	6 (27.3)	0.211
Hypertension, n (%)	15 (79.0)	15 (68.2)	0.499
Ischemic Heart Disease, n (%)	17 (94.7)	12 (54.6)	0.005
NYHA Functional Class III-IV, n (%)	2 (10.5)	8 (36.4)	0.075
Left Ventricular Ejection Fraction, median (IQR)	27% (22–34%)	30% (20%-47%)	0.638
VT storm, n (%)	3 (15.8)	5 (22.7)	0.703
Current Use of Anti-Arrhythmic Drugs, n (%)	15 (78.9)	18 (54.6)	0.562
Current Use of Amiodarone, n (%)	11 (57.9)	15 (45.5)	0.533
PAINESD, median (IQR)	12 (9–14)	12 (6–18)	0.522*
Impella 5.0/5.5, n (%)	3 (15.8)	4 (18.2)	1.000

* PAINESD score differ¬ence between groups was assessed around the population median (12)IQR = interquartile range; LV = left ventricle; NYHA = New York Heart Association; SD = standard deviation; VT = ventricular tachycardia

Among the 19 patients who underwent per-protocol parallel mapping, the majority were male (*n* = 18, 94.7%) with a mean age of 66 ± 10 years. VT storm was the presenting event in 3 patients (15.8%). The median PAINESD score was 12 (IQR 9–13). A temporary mechanical circulatory support with an Impella 5.0 or 5.5 device was preemptively implanted before the ablation in 3 patients (15.8%).

### Parallel mapping procedure

#### Procedural details

All patients underwent endocardial LV mapping, and 2 patients (9.1%) also underwent epicardial mapping. RV apical pacing was employed as the primary (S1) pacing wavefront in all cases (pacing and mapping strategies are summarized in Table 2; Supplemental Table 1 lists the different pacing wavefronts used for each scar distribution pattern). With respect to the secondary (S2) wavefront, left ventricular pacing was used in 10 (52.6%) patients (temporary LV pacing via a CS ventricular branch in 5; LV pacing via a permanent CRT system in 3; direct endocardial LV pacing in 2). Mapping during atrial pacing/sinus rhythm was employed in 7 (36.8%) patients (pacing from a temporary CS catheter in 5, pacing from a permanent right atrial lead in 1, and sinus rhythm in 1). RV outflow tract pacing was used in 1 (5.3%) patient. In one (5.3%) individual, the two parallel maps were generated during a pacing drive train (S1) and a single extra-stimulus (S2), all delivered from the same RV apical pacing catheter.

**Table 2:** Pacing strategies used for parallel mapping

Primary Wavefront (S1)	
RV Apical Pacing, n (%)	19 (100.0%)
Secondary Wavefront (S2)	
LV Pacing, n (%)	10 (52.6%)
Temporary Pacing via a Ventricular CS Branch, n (%)	5 (26.3%)
Pacing via a CRT Device, n (%)	3 (15.8%)
Direct Endocardial LV Pacing, n (%)	2 (10.5%)
Atrial Pacing / Sinus Rhythm, n (%)	7 (36.8%)
Temporary Pacing via CS, n (%)	5 (26.3%)
RA Pacing via a Permanent Atrial Lead, n (%)	1 (5.3%)
Sinus Rhythm, n (%)	1 (5.3%)
RV Outflow Tract Pacing, n (%)	1 (5.3%)
RV Apical Extra-Stimuli, n (%)	1 (5.3%)
Wavefront Alternation	
S1 ≥ 8 beats : S2 ≥ 8 beats, n (%)	9 (47.4%)
S1 < 8 beats : S2 < 8 beats, n (%)	4 (21.1%)
S1 5–8 beats : S2 ≤ 2 beats, n (%)	6 (31.6%)

CRT, cardiac resynchronization therapy; CS = coronary sinus; LV = left ventricle; RA = right atrium; RV = right ventricle

**Fig. 1:**
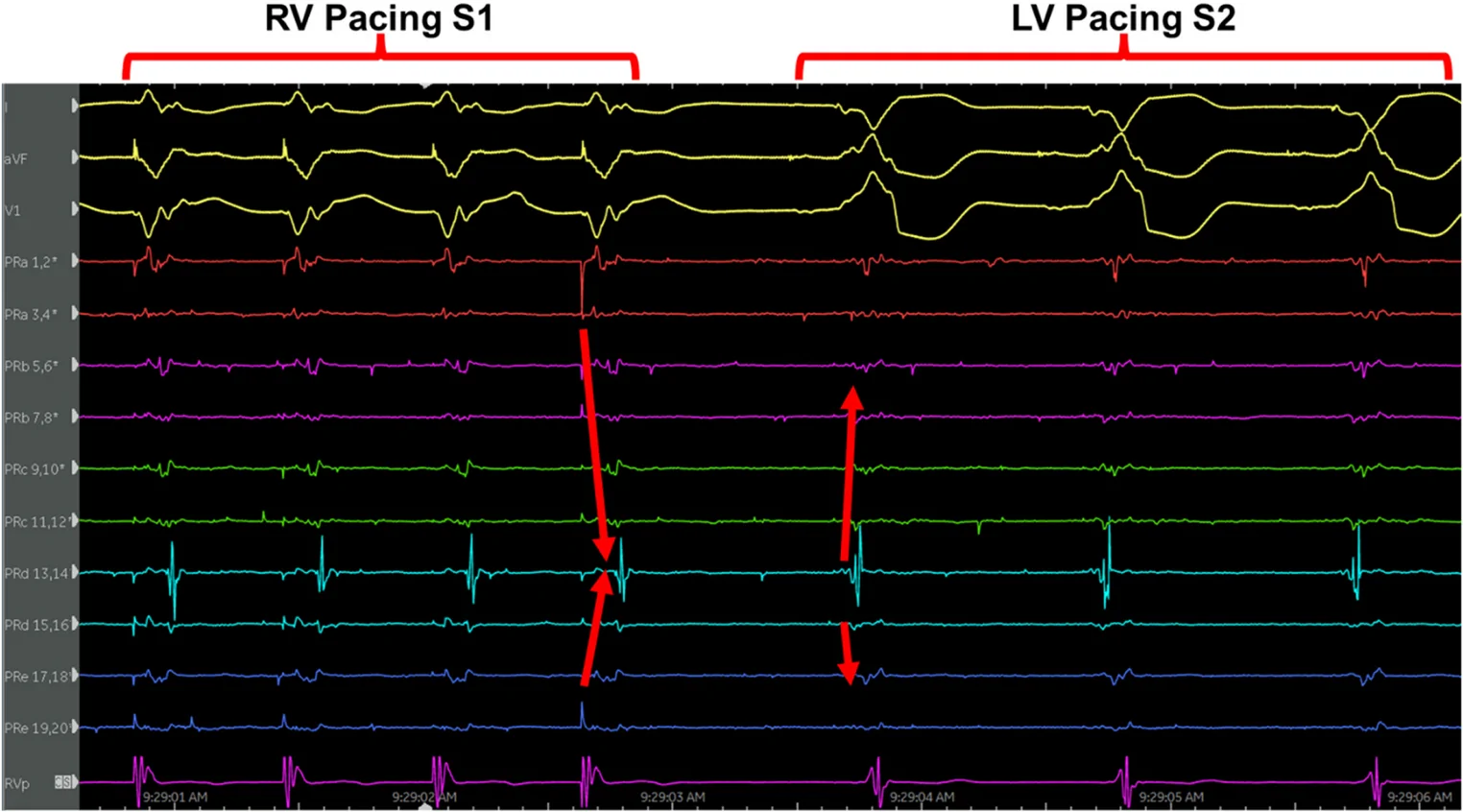
Example of wavefront alternation strategy during parallel mapping in a patient with an apical scar. Stimulation is delivered from a temporary pacing catheter in the RV (RV pacing S1) which alternates with LV pacing via an existing CRT device (LV Pacing S2). This resulted in a change in local activation pattern (arrows). CRT = cardiac resynchronization therapy; LV = left ventricle sinus; RV = right ventricle

During mapping, the pacing wavefronts were alternated using three different strategies. In 9 (47.4%) individuals, pacing from each wavefront was sustained for at least 8 beats before manually switching to the secondary waveform (i.e. S1 ≥ 8 beats : S2 ≥ 8 beats) using pacing cycle lengths > 500ms. The mapping catheter was roved continuously (while constantly collecting data), irrespective of the current pacing wavefront. In 4 (21.1%) patients, alternation between the two wavefronts was done automatically and more rapidly (i.e. S1 < 8 beats : S2 < 8 beats), also using pacing cycle lengths of > 500ms. In the context of this strategy, the operators attempted to obtain activation data from both wavefronts at each location before relocating the mapping catheter to another site (Fig. 1). In the remaining 6 (31.6%) patients, mapping was done using a short drive train (first waveform: S1 using 5–8 beats; pacing cycle length > 500ms) coupled to one or two extra-stimuli (second waveform: S2 ≤ 2 beats; median coupling interval 430ms [IQR 350-500ms]).

The median procedure duration was 252 min (IQR 190–289), and the median fluoroscopy time was 19 min (IQR 13–30). The median number of activation points per patient (i.e. primary and secondary maps combined) was 3,607 (IQR 2,198-4,045).

As expected, the use of single/double extra-stimuli to generate the secondary activation map resulted in lower point counts in that map compared to cases that employed more sustained pacing strategies (secondary map point count median 660 [IQR 347-1,070] vs. 1,311 [IQR 924-2,144], respectively, *p* = 0.023). However, the selection of mapping wavefront did not have a significant impact on the total number of collected points, mapping time or mapping rate (number of activation points collected per minute; Supplemental Table 2).

#### Correlation between the substrate to critical VT sites

Endocardial low-voltage areas were most commonly found in the inferior (*n* = 6, 31.6%), anterior (*n* = 4, 21.1%), and lateral (*n* = 5, 26.3%) walls.

RVZ regions were identified in all activation maps. In all (100%) cases, RVZ sites were entirely contained within areas of low bipolar voltage (≤ 0.5mV) or in their immediate vicinity (within ≤5 mm). VT was inducible in 18 subjects (94.7%) before ablation; VT was at least partially mappable before ablation in 14 patients (73.7%). In 12 of these cases (85.7%), the critical VT isthmus co-localized with reentry-vulnerable zones (RVZs) identified during parallel mapping. In 7 (58.3%) of these 12 cases, both activation maps were similar (i.e. no added value; both maps predicted the location of VT isthmus). In 4 (33.3%) cases, both parallel maps identified RVZs that correlated with VT isthmus, but one map yielded qualitatively more discrete information (i.e. clear isochronal crowding over a smaller area). RVZs were dramatically different between two maps only in 1 (8.3%) case (i.e. in this patient, RVZs from only one of the two maps clearly correlated with VT isthmus).

#### Post-procedural inducibility and adverse events

At the conclusion of the procedure, complete VT non-inducibility was achieved in 9 patients (47.4%). An additional 6 patients (31.6%) were inducible only for non-clinical VT, as assessed by the primary operator. In the remaining 4 patients (21.1%), no programmed ventricular stimulation was performed at the end of the procedure.

Procedure-related adverse events occurred in 4 patients (21.1%): 1 pericardial tamponade related to epicardial mapping/ablation requiring surgical repair; 1 reactive pericardial effusion managed conservatively; 1 episode of post-procedural cardiogenic shock with hypoxia requiring intubation followed by full recovery; and 1 femoral arterial hematoma requiring surgical intervention, which was followed by recurrent VT storm and cardiogenic shock, ultimately resulting in death. This mortality was attributed to VT storm rather than directly to the vascular complication (thus, this mortality was classified as possibly procedure-related).

Two patients (10.5%) were electively brought back to the EP lab for repeat ablation within 24 h of the index procedure; one of these cases required surgical epicardial access. The parallel mapping protocol was not used in these redo cases.

### Follow-up

**Fig. 2:**
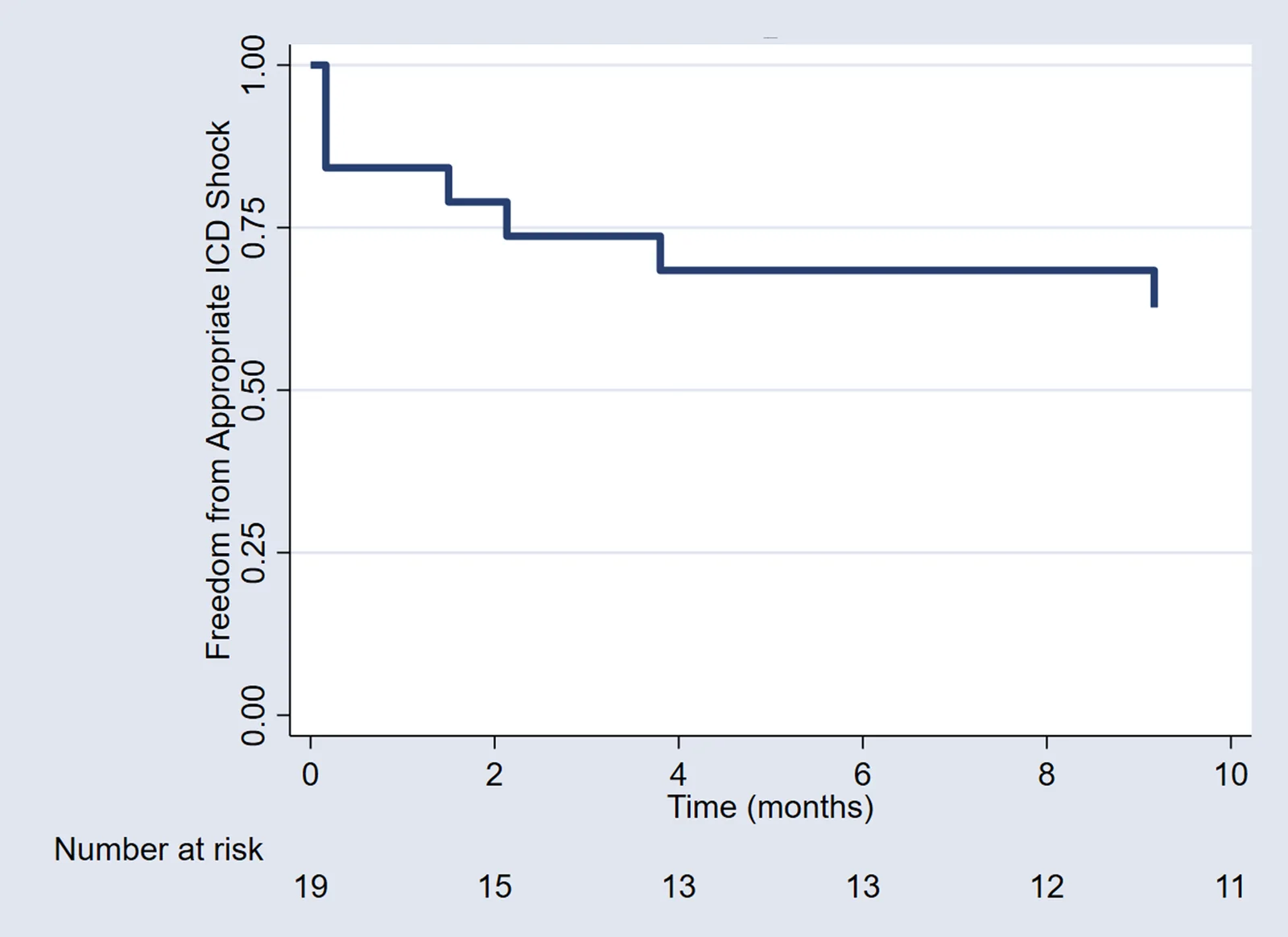
Post-ablation survival free from appropriate ICD shocks. ICD = Implantable Cardioverter-Defibrillator

During the 12-month active follow-up period (Fig. 2), one patient (5.3%) withdrew from the study. Seven (36.8%) individuals received an appropriate ICD shock and six (31.6%) patients underwent a redo VT ablation. Three (15.8%) patients died: 1 from primary pulmonary disease, 1 from end-stage heart failure and 1 from VT arrest. The median total follow-up time was 25.0 months (IQR 14.6–27.4 months).

### Exploratory analysis: insights from individual cases

#### Lateral wall substrate

Large lateral wall endocardial voltage abnormality was noted in 5 (26.3%) of patients. In four cases, adequate VT activation/entrainment mapping could be used to visually correlate RVZ areas with the site of putative VT isthmus.

**Fig. 3:**
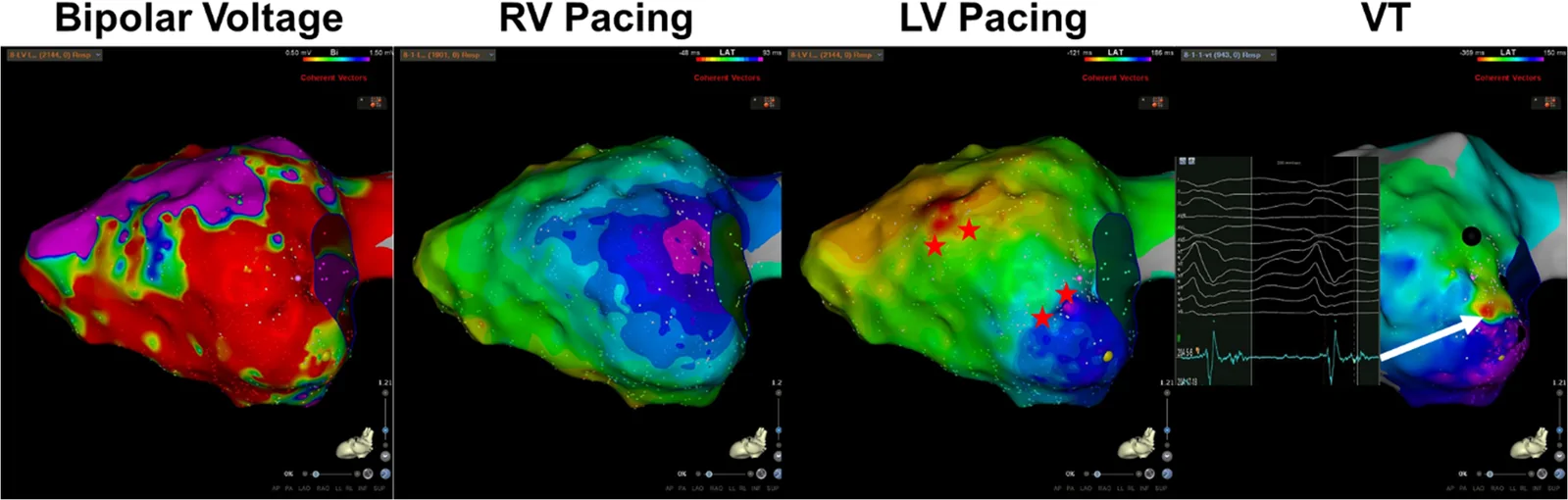
Example of lateral wall substrate (Patient 1). Bipolar voltage map revealed a large area of low-amplitude signals across the entire lateral wall. RV apical pacing produced a homogenous pattern of activation, while lateral LV pacing identified multiple re-entry vulnerable zones (stars), which correlated with a VT critical site. Bipolar voltage scale: 0.50-1.50mV. LV = left ventricle; RV = right ventricle; VT = ventricular tachycardia

Figure 3 illustrates an example of a patient with a large lateral wall scar, where parallel mapping produced markedly different RVZ distribution (Patient 1). Lateral LV wall pacing unmasked RVZs that later correlated with a critical VT site. After RVZ ablation, VT was no longer inducible and the patient had no appropriate ICD therapies during a 193-day follow-up (until death from end-stage heart failure).

In the other 4 patients with lateral scar (three with mappable VT that correlated with RVZ and one with no mappable VT), the location of RVZs was qualitatively similar between pacing wavefronts but wavefronts originating closer to the scar area produced RVZ maps that were easier to interpret (RV apical pacing better than RV outflow tract pacing; coronary sinus pacing/sinus rhythm better than RV apical pacing).

#### Anterior wall and apical substrate

Anterior wall/apical voltage abnormalities were identified in 4 (21.1%) patients, 2 of which had mappable VT circuits.

**Fig. 4:**
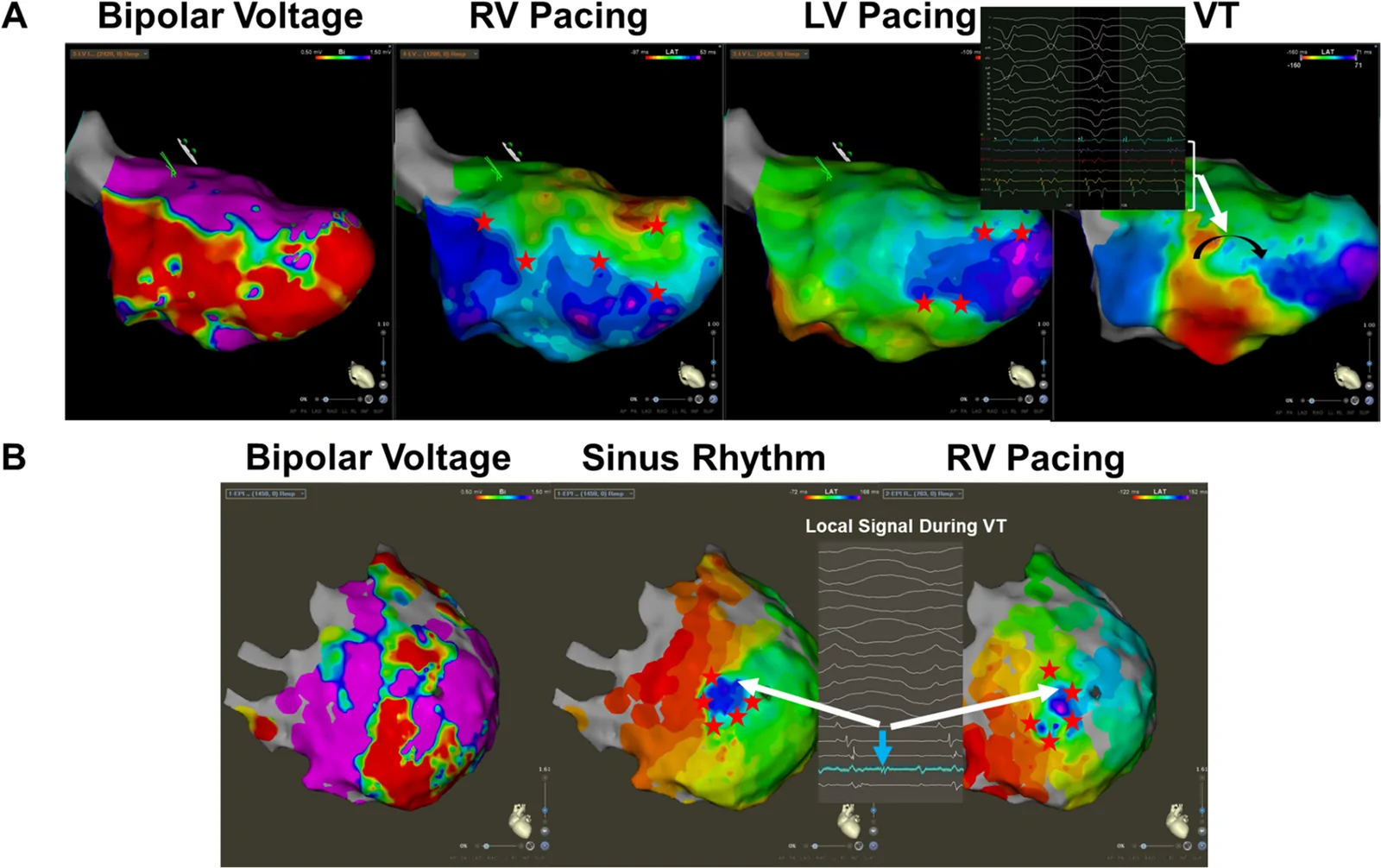
Examples of anterior wall/apical substrates. (**A**) Patient 2 had a large apical aneurysm that hosted RVZs (stars) identified during RV apical and lateral LV pacing; these correlated with the deduced VT critical site (black arrow; inset). (**B**) Patient 3 had a large anterior wall scar but endocardial maps showed no definite RVZs. Epicardial maps (shown) during sinus rhythm and RV pacing identified the same discrete RVZ locale (stars) that coincided with the location of mid-diastolic potentials during VT (inset; blue arrow). Bipolar voltage scale: 0.50-1.50mV. LV = left ventricle; RV = right ventricle; RVZ = re-entry vulnerable zone; VT = ventricular tachycardia

Figure 4 shows selected examples of these patients. Patient 2 (Fig. 4A) had a large apical aneurysm, with low voltage regions extending into the inferior wall. RV apical and lateral LV pacing revealed mutually complementary RVZ distribution that correlated with VT isthmus. Notably, RVZ distribution largely mirrored the borders of low bipolar voltage (< 0.5mV). RVZs were ablated and the patient had no sustained VT during the entire 1,282-day follow-up period.

In patient 3 (Fig. 4B), epicardial abnormalities dominated over endocardial abnormalities. Sinus rhythm and RV apical pacing maps resulted in similar epicardial RVZ distribution that, in turn, correlated with VT isthmus. However, this patient did experience sustained VT requiring an ICD shock 64 days after the procedure.

In the other 2 patients with anterior wall/apical scar (both with no mappable VT), RV apical and lateral LV pacing wavefronts produced mutually comparable results approximately correlating with the outline of the low bipolar voltage (< 0.5mV) scar.

#### Inferior wall substrate

Inferior wall voltage abnormalities were identified in 6 (31.6%) patients, 3 of which had mappable VT circuits.

**Fig. 5:**
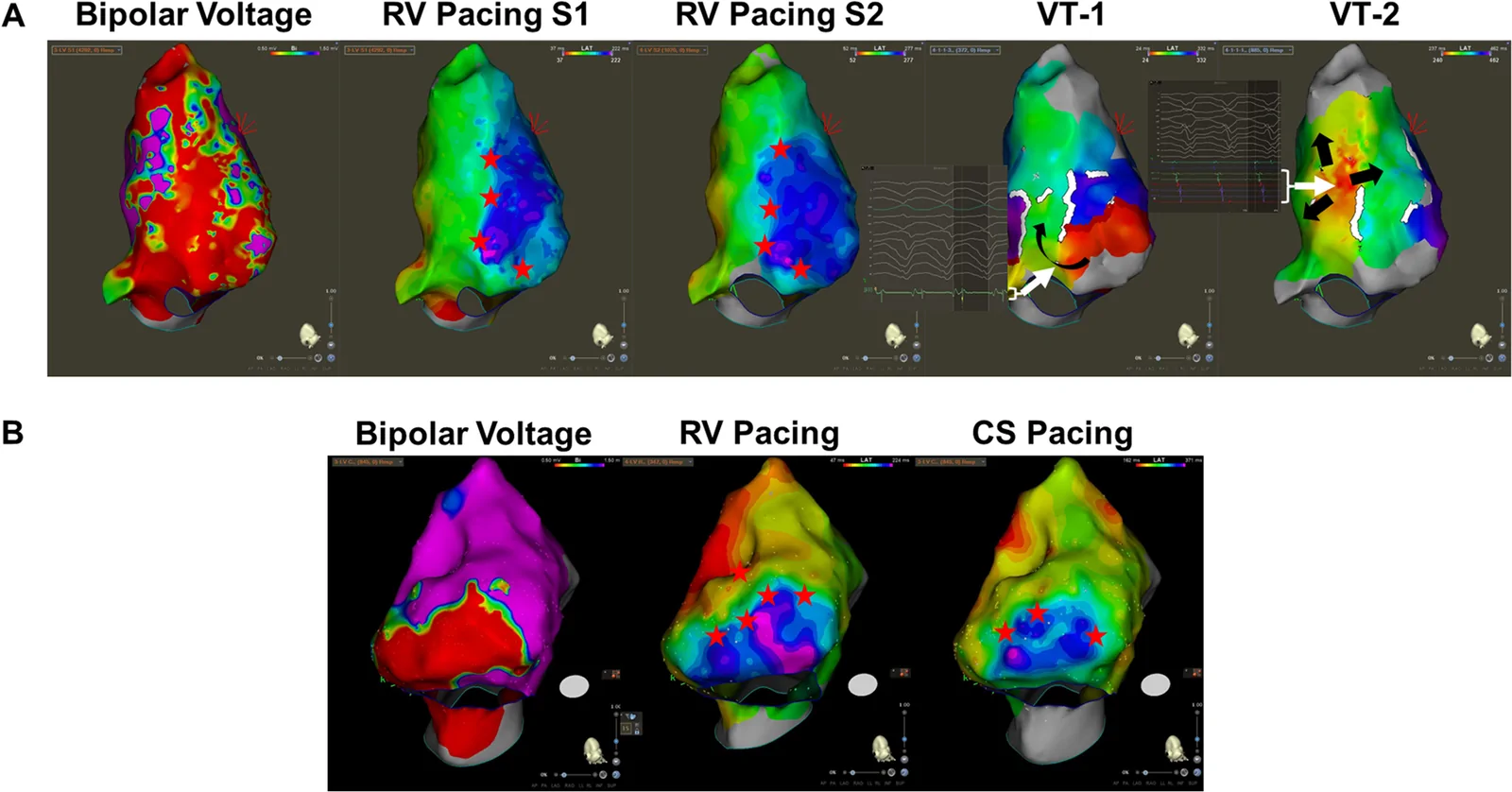
Examples of inferior wall substrates. (**A**) Patient 4 had a large inferior wall scar. Mapping during RV pacing at a constant cycle length (S1) and extra-stimuli (S2) revealed similar RVZ distributions (stars). These correlated with inferred critical VT sites (black arrows; insets). (**B**) The inferior wall voltage abnormality was less extensive in patient 5; again, multi-wavefront pacing from RV and CS produced similar RVZ maps (stars). Bipolar voltage scale: 0.50-1.50mV. CS = coronary sinus; LV = left ventricle; RV = right ventricle; RVZ = re-entry vulnerable zone; VT = ventricular tachycardia

Figure 5 shows selected examples of these patients. In patient 4 (Fig. 5A), RV pacing using constant cycle length (S1) and extra-stimuli (S2) produced similar RVZ distribution within a large inferior scar. These sites correlated with critical components of 2 separate VT circuits. After RVZ ablation, the patient remained ICD shock-free for > 2 years.

Patient 5 (Fig. 5B) exemplifies an inferior wall substrate mapped during wavefronts from 2 separate origins (RV apex and coronary sinus). Like in the case of patient 4, the RVZ distribution on these two maps was similar. RVZs were ablated and the patient remained ICD shock-free for > 2 years.

The 4 additional cases of inferior wall substrate (RV apical pacing/lateral LV pacing in 2 cases; RV apical pacing/atrial wavefront in 2 cases) also produced qualitatively similar RVZ maps for each pair of activation wavefronts.

#### Septal substrate

**Fig. 6:**
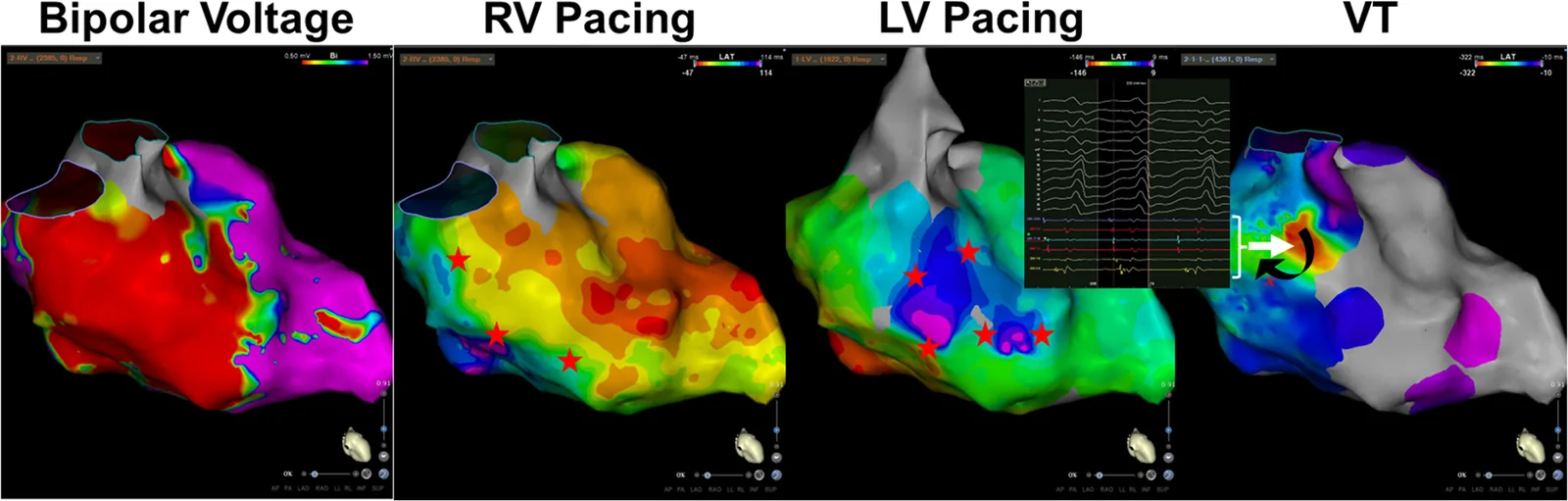
Example of septal substrate. Patient 6 had a very large bipolar voltage abnormality that spanned the entire basal septum. Multi-wavefront pacing produced a chaotic distribution of RVZs (stars) that did little to narrow down the choice of ablation targets. VT (black arrow; inset) was mapped to the basal septum. Bipolar voltage scale: 0.50-1.50mV. LV = left ventricle; RV = right ventricle; RVZ = re-entry vulnerable zone; VT = ventricular tachycardia

Septal voltage abnormalities were identified in 3 (15.8%) patients, 1 of which had a mappable VT circuit. All 3 patients were mapped using RV apical and LV lateral pacing wavefronts. The distribution of RVZs differed with each pacing wavefront; however, RVZs spanned much of the septum in all cases. For example, the activation maps in patient 6 (Fig. 6) did little to further our understanding of the septal VT circuit.

#### Diffuse substrate

One patient (5.3%) exhibited abnormal bipolar voltage throughout the LV; multiple VTs were inducible including a perimitral VT (with an inferior exit) and another VT originating from the mid-anterior wall. Activation maps obtained during RV apical pacing and sinus rhythm showed no clear endocardial RVZs.

## Discussion

In this pilot study, the use of the parallel mapping algorithm proved to be feasible in 46.3% of enrolled patients slated for catheter ablation of scar-related VT. Patients in whom parallel mapping could not be completed were more likely to suffer from nonischemic cardiomyopathy. Conversely, the protocol was most consistently feasible in individuals with ischemic cardiomyopathy. Procedural duration, radiation exposure, procedural complications and long-term outcomes in patients who underwent per-protocol parallel mapping-guided ablation were comparable to benchmark data. In exploratory analysis, parallel mapping during two different activation wavefronts appeared to provide most informative diagnostic data in patients with a lateral wall scar.

The concept that zones of slow conduction may herald the presence of arrhythmogenic abnormalities in functional myocardial substrate is not new [8, 9]; however, Aziz et al. [5] were first to show that targeting such zones for ablation was a viable means of treating VT. Similar findings were replicated by the PHYSIO-VT study [6] just one year later.

However, the relationship between the location and significance of RVZs on one hand and the activation wavefront during which mapping was done on the other hand is less clear. In the PHYSIO-VT study, the use of more than one activation wavefront unmasked 33% RVZs, correlating with 25% more VT isthmus sites compared to single wavefront mapping. Indeed, multi-wavefront mapping was associated with improved freedom from appropriate ICD therapies during follow-up. In a recent in silico study, the potential benefit of multi-wavefront functional assessment was extended to include DEEP mapping [10]. 

Challenges to additional research in this field include the time-consuming and labor-intensive nature of sequential multi-wavefront mapping. Fortunately, if feasible in this context, the parallel mapping algorithm may help overcome this barrier by streamlining procedural workflow.

The present study shows that parallel multi-wavefront mapping of scar-related VT substrate is, indeed, feasible. Although over half (53.7%) of enrolled patients did not end up undergoing per-protocol mapping, this finding frequently reflected a broader clinical decision to forgo all forms of substrate mapping (36.6% of enrolled patients were only mapped/ablated during VT or were found to have no scar-related substrate). Actual attempts at multi-wavefront parallel mapping were made and abandoned in only 17.1% of enrolled patients – frequently because of high burden of mapping catheter-related ectopy. Presence of NICM substrate was the only significant predictor of deviation from the parallel mapping protocol, which is not surprising given this population’s anecdotally higher susceptibility to catheter-induced ectopy.

With a median procedure time of 252 min (IQR 190–289 min) and median fluoroscopy time of 19 min (13–30 min), the parallel mapping procedure did not noticeably deviate from published studies (e.g. VANISH2 procedure time of 212 min [IQR 168–280 min]/fluoroscopy time of 11 min [IQR 6–17 min]; PAUSE-SCD procedure time of 240 min [IQR 166–280 min]) [11, 12]. 

The rate of procedure-related adverse events (4 cases, 21.1%) was higher than the published data in unstratified patient populations [13]. However, 3 complications were related to access (2 epicardial access-related complications; 1 femoral arterial access-related complication). Only one (5.3%) patient developed immediate post-procedural cardiogenic shock, a complication that could conceivably have been related to the parallel-mapping protocol. This relatively elevated rate of complications may reflect the high risk-nature of the patient population, 16.8% of whom required intraprocedural mechanical hemodynamic support; indeed, complication rates in such population are often very high [14, 15]. One-year VT recurrence rate in our study was also comparable to published data.

With data available from only 19 per-protocol treated patients, no definitive conclusions can be drawn about the interplay between scar distribution, selection of activation wavefronts and outcomes. However, a few salient observations can be carefully stated. First, parallel mapping was subjectively most informative in patients with lateral wall substrate; in all 5 of these individuals, the RVZ maps generated based on 2 pacing wavefronts were very different and thus likely mutually complimentary. However, in some instances, this observation may simply highlight the limitations of mapping during RV apical pacing which produces very late activation in the basal lateral wall; virtually any other pacing wavefront may thus be better equipped to identify RVZs in this segment.

Second, parallel mapping produced subjectively least valuable data in patients with septal substrates. Predominantly septal distribution of endocardial voltage abnormalities is uncommon in ischemic cardiomyopathy. This raises the possibility that the 3 patients in this study who primarily exhibited septal scarring may have suffered from a “mixed” form of cardiomyopathy. Since septal VT substrate may be entirely intramural, it is not surprising that endocardial RVZ assessment may be of little use [16]. Alternative methods for septal substrate identification may hold more promise in this setting [17]. 

It is also worth noting that multi-wavefront mapping is not limited to the parallel mapping algorithm discussed in this study. For example, other software packages (such as the TurboMap feature of the EnSite Precision mapping system, Abbott, Abbott Park, IL) allow for *post hoc* acquisition of mapping data generated using multiple pacing wavefronts.

### Study limitations

This single-arm pilot study was designed to test the feasibility of multi-wavefront parallel mapping algorithm in VT ablation. Thus, no definitive conclusions can be made about this technique’s effect on procedural parameters (such as mapping times) or on patient outcomes. Furthermore, there was an under-representation of patients with NICM in this study as most NICM patients were excluded from this investigation due to lack of identifiable electrical substrate or due to a clinical decision even after study enrollment. Additionally, per-protocol parallel mapping was only used in 46.3% patients and (other than presence of NICM), we could not identify any a priori markers for individuals in whom this protocol ultimately was not feasible. Epicardial substrate parallel mapping was also done only in 2 patients, which limits the generalizability of our observations. Additionally, the assortment of the pacing protocols and pacing wavefronts employed in each case (which were chosen based on individual operator’s discretion) along with the heterogeneity of the myocardial substrate of each study subject (e.g. varying size and location of infarct) prevents us from making any definitive, statistically tested statements about the utility of each parallel mapping strategy in each clinical context.

Of note, the use of mechanical hemodynamic support in study subjects was arbitrary and reflected the risk of acute hemodynamic decompensation as perceived by the operator; this may have biased our data.

In some cases, induced VTs were thought to be clinically irrelevant by the operator and were not mapped; this adjudication of clinical relevance was entirely subjective and may have introduced a sampling bias into the study. Furthermore, operators may have been biased to focus their mapping efforts on areas with most obvious voltage abnormalities and RVZs in other regions may have gone unrecognized regardless of which parallel mapping strategy was employed. Some of these limitations could be addressed in future studies by mandating stricter inclusion criteria and more homogenous pacing and mapping strategies.

## Conclusions

The parallel mapping algorithm is a feasible means of assessing scar-related myocardial VT substrate, especially in patients with ischemic cardiomyopathy. The algorithm may have a more limited feasibility in certain groups, such as those with a nonischemic substrate or those susceptible to frequent catheter ectopy. Procedural duration, radiation exposure, complications and outcomes are comparable to benchmark data. Parallel mapping may have highest clinical utility in patients with lateral wall substrate; however, additional studies are needed to confirm this technique’s performance and impact on outcomes across all VT substrate types. Studies are also needed to directly compare the feasibility and utility of parallel mapping technique to single wavefront mapping.

## Supplementary Material

Below is the link to the electronic supplementary material.

Supplementary Material 1 (17.4 KB DOCX)

## Data Availability

The data that support the findings of this study are not openly available due to reasons of sensitivity and are available from the corresponding author upon reasonable request.

## Abbreviations

CS: Coronary Sinus
DEEP: Decremental Evoked Potential
ICD: Implantable Cardioverter-Defibrillator
IQR: Interquartile Range
LVEF: Left Ventricular Ejection Fraction
LV: Left Ventricle
NICM: Non-ischemic Cardiomyopathy
NYHA: New York Heart Association
RFA: Radiofrequency Ablation
RV: Right Ventricle
RVZ: Re-entry Vulnerable Zone
SD: Standard Deviation
VERP: Ventricular Effective Refractory Period
VF: Ventricular Fibrillation
VT: Ventricular Tachycardia

## Declarations

### Trial Registration Number

NCT04477499 (clinicaltrials.gov database).
