# Supplementary material for: PARALLEL-VT: A pilot study of simultaneous multi-wavefront left ventricular mapping for identification of critical ablation sites
Source: J Interv Card Electrophysiol. 2026 May 6;69(8):2399–409. doi: 10.1007/s10840-026-02347-1 (PMC13740072; doi:10.1007/s10840-026-02347-1)
Supplement: Supplementary file 1 [file 10840_2026_2347_MOESM1_ESM.docx]

**Supplemental Material**

The following Micropace EP stimulator settings were used during parallel mapping.

In all cases, the “MULTI_SX” pacing protocol was used. The desired number of S1 pacing stimuli were delivered at a fixed cycle length from the RVA catheter (Channel 3).

In situations where the secondary wavefront was generated extrinsically with respect to the Micopace stimulator (i.e. sinus rhythm, atrial/ventricular pacing using a permanent implantable device), the S1 drive train was followed by a programmed pause. Pause duration tailored to the desired number of extrinsic S2 beats.

In situations where the secondary wavefront was generated intrinsically with respect to the Micropace stimulator (i.e. S2 delivered from the RVA catheter or another temporary pacing catheter, Channels 1-2), this was specified in the MULTI_SX protocol settings. The MULTICHAN function allows specification of how many stimuli are delivered to each channel. When using short-coupled S2/S3 extra-stimuli, a 2,000-3,000ms pause was used.

**Supplemental Table 1**

| **Scar Distribution** | **Secondary Wavefront** |
| --- | --- |
| Anterior | Atrial, n=1 |
|  | Left ventricular, n=1 |
| Apex | Left ventricular, n=2 |
| Inferior | Atrial, n=1 |
|  | Left ventricular, n=4 |
|  | Right ventricular (apex, extra-stimuli), n=1 |
| Lateral | Atrial, n=4 |
|  | Left ventricular, n=1 |
|  | Right ventricular (outflow tract), n=1 |
| Septum | Atrial n=1 |
|  | Left ventricular, n=2 |

**Supplemental Table 1:** List of pacing wavefronts used for each scar distribution pattern. Primary wavefront was delivered from the right ventricular apex in all cases.

**Supplemental Table 2**

|  | **LV Activation Used as Secondary Wavefront (S2), n=10** | **All Other Secondary Wavefronts (S2), n=9** | **P-value** |
| --- | --- | --- | --- |
| **Total Point Count, median (IQR)** | 3,636 (2,198-4,007) | 2,670 (2,213-4,990) | 0.807 |
| **Total Mapping Time, minutes, median (IQR)** | 47.5 (43.0-58.0) | 42.0 (28.0-47.0) | 0.269 |
| **Mapping Rate, points per minute, median (IQR)** | 70.8 (58.5-84.3) | 90.3 (58.8-147.5) | 0.414 |
|  | **Atrial Activation Used as Secondary Wavefront (S2), n=12** | **All Other Secondary Wavefronts (S2), n=7** | **P-value** |
| **Total Point Count, median (IQR)** | 2,670 (1,370-4,990) | 3,636 (2,364-4,026) | 0.499 |
| **Total Mapping Time, minutes, median (IQR)** | 42.0 (28.0-64.0) | 46.5 (37.5-53.0) | 0.525 |
| **Mapping Rate, points per minute, median (IQR)** | 79.0 (32.6-178.2) | 80.8 (60.0-99.7) | 0.866 |
|  | **Secondary Wavefront Generated Using 1-2 Extra-Stimuli (S2 1-2 beats), n=6** | **All Other Secondary Wavefronts (S2≥beats), n=13** | **P-value** |
| **Total Point Count, median (IQR)** | 2,958 (1,370-4,990) | 3,620 (2,213-4,007) | 0.661 |
| **Total Mapping Time, minutes, median (IQR)** | 44.0 (28.0-47.0) | 47.0 (32.0-58.0) | 0.404 |
| **Mapping Rate, points per minute, median (IQR)** | 86.7 (32.6-147.5) | 79.0 (62.4-90.3) | 0.861 |

**Supplemental Table 2:** Comparison of Pacing/Mapping strategies and their impact on procedural parameters. Data here reflect per-patient totals, i.e. point counts, mapping times and mapping rates used to generate both the primary and the secondary maps in each patient. IQR = interquartile range; LV = left ventricle.
